# GWASdb v2: an update database for human genetic variants identified by genome-wide association studies

**DOI:** 10.1093/nar/gkv1317

**Published:** 2015-11-28

**Authors:** Mulin Jun Li, Zipeng Liu, Panwen Wang, Maria P. Wong, Matthew R. Nelson, Jean-Pierre A. Kocher, Meredith Yeager, Pak Chung Sham, Stephen J. Chanock, Zhengyuan Xia, Junwen Wang

**Affiliations:** 1 Centre for Genomic Sciences, LKS Faculty of Medicine, The University of Hong Kong, Hong Kong SAR, China; 2 School of Biomedical Sciences, LKS Faculty of Medicine, The University of Hong Kong, Hong Kong SAR, China; 3 Department of Anaesthesiology, LKS Faculty of Medicine, The University of Hong Kong, Hong Kong SAR, China; 4 Department of Pathology, LKS Faculty of Medicine, The University of Hong Kong, Hong Kong SAR, China; 5 Quantitative Sciences, GlaxoSmithKline, Research Triangle Park, NC, USA; 6 Division of Biomedical Statistics and Informatics, Mayo Clinic College of Medicine, Rochester, MN, USA; 7 Division of Cancer Epidemiology and Genetics, National Cancer Institute, NIH, Bethesda, MD, USA; 8 State Key Laboratory of Brain and Cognitive Sciences and Department of Psychiatry, LKS Faculty of Medicine, The University of Hong Kong, Hong Kong SAR, China

## Abstract

Genome-wide association studies (GWASs), now as a routine approach to study single-nucleotide polymorphism (SNP)-trait association, have uncovered over ten thousand significant trait/disease associated SNPs (TASs). Here, we updated GWASdb (GWASdb v2, http://jjwanglab.org/gwasdb) which provides comprehensive data curation and knowledge integration for GWAS TASs. These updates include: (i) Up to August 2015, we collected 2479 unique publications from PubMed and other resources; (ii) We further curated moderate SNP-trait associations (*P*-value < 1.0×10^−3^) from each original publication, and generated a total of 252 530 unique TASs in all GWASdb v2 collected studies; (iii) We manually mapped 1610 GWAS traits to 501 Human Phenotype Ontology (HPO) terms, 435 Disease Ontology (DO) terms and 228 Disease Ontology Lite (DOLite) terms. For each ontology term, we also predicted the putative causal genes; (iv) We curated the detailed sub-populations and related sample size for each study; (v) Importantly, we performed extensive function annotation for each TAS by incorporating gene-based information, ENCODE ChIP-seq assays, eQTL, population haplotype, functional prediction across multiple biological domains, evolutionary signals and disease-related annotation; (vi) Additionally, we compiled a SNP-drug response association dataset for 650 pharmacogenetic studies involving 257 drugs in this update; (vii) Last, we improved the user interface of website.

## INTRODUCTION

Ten years’ efforts on genome-wide association study (GWAS) have produced large numbers of human genetic variants that are associated with hundreds of medical traits and diseases. The world of GWAS is evolving rapidly with improved technologies such as high-density genotyping array and next generation sequencing (NGS) (1,2). New directions of GWAS are mostly focused on larger sample size (3), variants fine-mapping (4), meta-analysis (5), cross-phenotype association (6), sequencing-based test (7), etc. These strategies are increasingly employed to pinpoint the full spectrum of common, low frequency even rare variants that potentially contribute to human traits and disorders. A recent study showed that genetic evidence from GWASdb (8) or OMIM (9) have the potential to double the success rate of drug development (10), indicating that GWAS is moving from basic science to translational applications.

Although GWAS Catalog (11) and other databases, such as PheGenI (12), GWAS Central (13), SNPedia (14), and GRASP (15), have collected significant trait/disease associated SNPs (TASs) at different levels, comprehensive curation and function annotation of TASs, especially for those in the noncoding regulatory regions, are still lacking. The Encyclopedia Of DNA Elements (ENCODE) project (16) and Roadmap Epigenomics Project (17) have successfully identified many functional elements and regulatory units in the human genome. Meanwhile, different bioinformatics tools have been developed to predict SNP functions in multiple biological domains (18–20). Unfortunately, very few GWAS resources have incorporated these information to annotate TASs. In addition, GWAS usually utilizes natural language to describe investigated traits. The inconsistent terms used for similar or even identical traits prevent easy comparison and analysis among different GWASs. In spite that GWAS Catalog, GWAS Central and previous publication version of GWASdb have mapped many GWAS trait/disease descriptions to Experimental Factor Ontology (EFO) (21), Medical Subject Headings (MeSH) (22) and Human Phenotype Ontology (HPO) (23), efforts are needed for continuous integration of up-to-date ontology information and GWAS traits.

In this update, we systematically collected TASs, as well as detailed information for their effect size and investigated population, from published GWASs. We performed deep and high-quality curation for moderate effect TASs according to related materials of each publication. GWASdb v2 also introduces a batch of new features including new ontology mapping, multi-level annotation, causal gene prediction and an updated interactive user interface.

## MATERIALS AND METHODS

### Data curation and collection

We collected all significant TASs (*P*-value < 1.0 × 10^−5^) from GWAS Catalog, PheGenI and HuGE (24). Due to the omissions and different curation standards for these major resources, we also searched GWAS publications from PubMed using key words such as ‘Genome-wide association’, ‘genome association’ (Supplementary Methods). To reduce data redundancy, we first collected GWAS Catalog data, and then excluded those overlapped data when integrating variants from other datasets and our curation (Supplementary Methods).

Similar to last version, we systematically curated moderately significant TASs from related documents of each original GWAS publication. Generally, we collected TASs by using a *P*-value of less than 1.0 × 10^−3^, since many susceptible loci may only show moderate significance in association analysis. Variants were extracted from both full text and supplementary materials following our criteria (Supplementary Methods). In GWASdb v2, we further added reported effect sizes to characterize SNP-trait associations besides of *P*-values, such as odds ratio/beta, 95% CI information, text remark of *P*-value, and risk allele. For each GWAS, in addition to its PubMed ID or Analysis ID (studies from PheGenI), we also provide detailed annotation on sample size and ancestry of initial stage if recorded (known as sub-populations in GWASdb v2). Then, we categorized these sub-populations into eight ethnogeographic super-populations, namely European/Caucasian (EUR), African (AFR), East Asian (ASN), Native American (AMR), Hispanic/Latino (HIS), Middle Eastern (MEA), South Asian (SAN), Oceania (OCN) and ambiguous samples (OTHER), and recorded the corresponding sample size information (Supplementary Methods).

### Ontology mapping

The inconsistence of original trait/disease descriptions from different GWASs impedes the large scale integrative analysis among independent studies. For example, different studies utilized distinct phenotype descriptions for an identical disease (e.g. ‘HIV-1 viral setpoint’ and ‘HIV mother-to-child transmission’ are both HIV related) or several studies involved measurement of particular molecules which are well-recognized biomarkers or risk factors for certain diseases (e.g. ‘Lipoprotein A level’ as a risk factor for coronary heart disease). Meanwhile, ontologies provide a computer friendly structure for semantic integration of biomedical diseases and phenotype terminologies. Therefore, a high quality mapping of natural language descriptions to formal and consistent ontology systems is an essential but challenging task. To this end, we used in-house software MapIn (http://jjwanglab.org/mapin/, unpublished), which could calculate the similarities between strings, to map various trait/disease descriptions from different GWASs to several well-defined ontology systems, including Disease Ontology (DO) (25), Human Phenotype Ontology (HPO), and Disease Ontology Lite (DOLite) (26). After automatic mapping, we manually checked each result to correct error mapping results and unmapped phenotypes (Supplementary Methods).

### Causal gene prediction

In this update version, we also provided causal gene prediction for each ontology trait/disease using a recent algorithm PrixFixe (27). Causal genes are usually predicted based on the closest genomic distance between TASs and genes, and the candidate prioritization tends to be biased towards well-studied genes (27). To overcome these obstacles, PrixFixe utilized a human co-function network to identify functionally related genes within GWA loci. In GWASdb v2, for each ontology trait/disease, we first ranked all the SNPs by *P*-values, then selected the top 200 (commonly used number in PrixFixe) SNPs to perform causal gene prediction. The exact SNP number was used if it is <200 for a certain trait/disease (Supplementary Methods).

### SNP-drug response dataset collection

Genetic polymorphisms may determine individual variability in drug response (28). Detection of correlation between SNPs and drug response is of great importance for personalized medicine. To this end, we compiled an independent SNP-drug response dataset. The data were collected from (i) GWASs which have been reported as drug-related studies in GWASdb v2; (ii) DIYgenomics Drug Response (http://www.diygenomics.org/webapp/pharma_data.php), which reports variants for 200 drugs from various references. Drugs were defined according to records from DrugBank (29) (Supplementary Methods).

### Data processing for annotation

For each TAS, we first mapped it to dbSNP142 and obtained the allele information from the 1000 Genomes Project (Supplementary Methods). We retrieved correlated SNPs in linkage disequilibrium (LD) with this TAS based on eleven HapMap I+II+III populations or four super populations from 1000 Genomes Project. Gene and genomic element data were downloaded from UCSC and GENCODE. We utilized ENCODE data to annotate the functional elements at the SNP position. Since GWAS TASs could map to genomic regions that are responsible for distinct biological functions, we also used sophisticated software to predict the functional consequences for different types of TASs across different biological domains, including transcription factor binding and gene regulation (GWAS3D (30)), microRNA–target interaction (PolymiRTS (31)), splicing (Skippy (32) and MutPred Splice (33)), non-synonymous variant in protein function (dbNSFP (34)), etc. Evolutionary information were also borrowed to annotate variants under different natural selection, such as positive selection scores (dbPSHP (35)) and conservation (PhyloP (36) and GERP++ (37)). For disease-related annotation, we collected genetic evidence information from OMIM, GAD (38), ClinVar (39) and COSMIC (40) (Supplementary Table S2).

### Database design

Compared with the previous GWASdb version, we improved the GWASdb v2 architecture by combining jQuery plugins (such as Highcharts, DataTables and related UI frontend) with a Perl-based web framework Catalyst. Annotation information were either stored in MySQL database or flat files indexed by Tabix (41). We used Circos (42) to generate global GWASdb v2 SNPs Manhattan plot and personal genome browser (PGB) (43) to display important annotation tracks.

## RESULTS

### Summary of new features

GWASdb v2 significantly extends the data content by deep manual curation and comprehensive resources integration. Compared with existing databases, GWASdb v2 covers the highest number of GWAS publications in the field (Supplementary Table S3). The extensive data volume for moderate effect SNPs will facilitate the finding of more associations that imply important biological function. Also, we updated well-organized trait/disease-ontology mappings including HPO, DO and DOLite, which will assist an effective trait organization. For mapped ontology terms, GWASdb v2 provides putative causal genes. Moreover, GWASdb v2 now clearly classifies each GWAS to respective sub- and super-populations, which will benefit researchers in studying population-specific traits. In addition, GWASdb v2 has collected a SNP-drug response dataset which could potentially benefit for pharmacogenetic research. Last but not least, GWASdb v2 compiles a complete annotation in both interactive web pages and local datasets. These annotation and visualization functions will help users pinpoint the functional attributes of TASs. Detailed improvements in GWASdb v2 since last publication version is shown in Supplementary Table S4.

### Statistics of GWASdb v2

Based on August 2015 version of GWASdb v2, 2479 unique GWASs have been collected and curated, which totally included 297 670 SNP-trait/disease associations (40 248 reached genome wide significance level with *P*-value < 5.0 × 10^−8^ and 257 422 had the moderate effect size with *P*-value < 1.0 × 10^−3^). Apart from SNPs collected from existing GWAS resources (GWAS Catalog, HuGE, and PheGenI, Supplementary Table S1), GWASdb v2 further curated 266 338 TASs by ourselves. GWASdb v2 contained 252 530 unique TASs. Among them, about 17% were reported by more than one cohort (i.e. different populations) and over 21% were associated with more than one trait/disease (according to DOLite mapping results), suggesting that many GWAS SNPs have shared association across human populations and are relevant to multiple genetic traits. Also, we observed an unbalanced distribution (Supplementary Figure S1) for the number of TASs in three major human populations (EUR, AFR and ASN) although current GWASs tend to investigate more worldwide populations. Majority of TASs are associated with several top investigated traits/diseases (according to DO or HPO mapping results, Supplementary Figure S2), including nervous system disorders (e.g., Parkinson's disease, Alzheimer's disease, and bipolar disorder), metabolic disorders (e.g., type 2 diabetes and obesity), cardiovascular diseases (e.g. myocardial infarction, hypertension, and arteriosclerosis) and immunological diseases (e.g. Systemic lupus erythematosus and lymphoma). The genomic distribution of GWASdb v2 TASs (Supplementary Figure S3) showed that 105 893 of them locate in the intergenic region and most of remaining genic TASs (92.8%) come from intronic region, indicating the regulatory role of these non-coding genetic variants.

In GWASdb v2, we were able to map 88% of variants to DO, 99% of variants to HPO, and 87% of variants to DOLite. We also collected and reported the EFO mapping from GWAS Catalog and the MeSH mapping from previous publication (10). These mapping repositories offer the largest GWAS phenotype-ontology resources (Supplementary Table S3). Additionally, 650 pharmacogenetic studies were collected with 524 studies from DIYgenomics Drug Response and 126 studies from GWASdb v2. In the current dataset, 257 drugs including FDA-approved (∼93%) and experimental (∼3%) drugs were recorded.

### Annotation of GWASdb v2 TASs

We utilized over 40 different datasets and prediction tools to annotate all GWASdb v2 TASs (Table 1), including gene-based information, knowledge-based information, biological function prediction across multiple domains, evolutionary signals and disease-related evidence. GWASdb v2 provides both interactive annotation web pages and downloadable annotation files for each TAS. According to the functional prediction of GWAS leading variants that achieved genome wide significance level, we found 12.1% TASs are predicted to affect at least one biological domain including transcription factor binding, alternative splicing, miRNA-target recognition, protein-function alternation, and protein phosphorylation. Compared with dataset randomly drawn from dbSNP 142 (3.3% have predicted effects), current GWASdb v2 TASs are significantly enriched in affecting biological functions (*P* < 2.2 × 10^−16^, Pearson's chi-squared test). This result is consistent with previous studies that GWAS SNPs are enriched in functional elements (44), eQTL (45) and positive selection signal (46).

**Table 1.** Annotation items of GWASdb v2

	SNP information	Gene-based annotation	Knowledge-based annotation	Functional prediction	Evolution annotation	Disease annotation	External link
Annotations	Manually curated (250k), dbSNP 142, 1000G phase I, HapMap and 1000G LD	RefGene, EnsembleGene, KnownGene, GENCODE, Small RNA, MicroRNA target sites	Validated and predicted enhancer, Insulator, HapMap and GTEx eQTL, Long range interaction (5C, ChIA-PET, Hi-C), ENCODE ChIP-seq, ENCODE functional elements	Transcriptional factor binding site affinity, MicroRNA target site affinity, Splicing site affinity, Non-synonymous SNP functional prediction, Synonymous SNP functional prediction, Phosphorylation site functional prediction	Positive selection, Conserved functional RNA, PhastCons, GERP++ elements	OMIM, ClinVar, Cosmic, DGV, GAD	SNPedia, Regulomedb, HaploReg, rSNPBase, UCSC Genome Browser, GWAS central

*Note*: 1000G: 1000 Genomes Project; HapMap: The International HapMap Project; ENCODE: Encyclopedia Of DNA Elements; 5C: Carbon-Copy Chromosome Conformation Capture; ChIA-PET: Chromatin Interaction Analysis by Paired-End Tag Sequencing; DGV: Database of Genomic Variants; GAD: Genetic Association Database.

### Comparison with existing resources

Since different GWAS resources follow different SNP collection criteria (e.g. various *P*-value threshold), it is unfair to directly compare the data volume with them. We alternatively compared the database features in different aspects (Supplementary Table S3). In general, the strengths of GWASdb v2 lie in the following aspects: (i) GWASdb v2 is the largest resource that collects the most GWAS publications; (ii) GWASdb v2 provides manually curated and high-quality SNPs which have less significance but potentially association effects; (iii) GWASdb v2 supports a couple of useful and comprehensive embedded functions (ontology mapping, causal gene prediction, annotation, drug response dataset, visualization, web services, etc). Therefore, to the best of our knowledge, GWASdb v2 is the most comprehensive database in the GWAS community.

### Usage of the GWASdb v2 web interface

**Figure 1.**
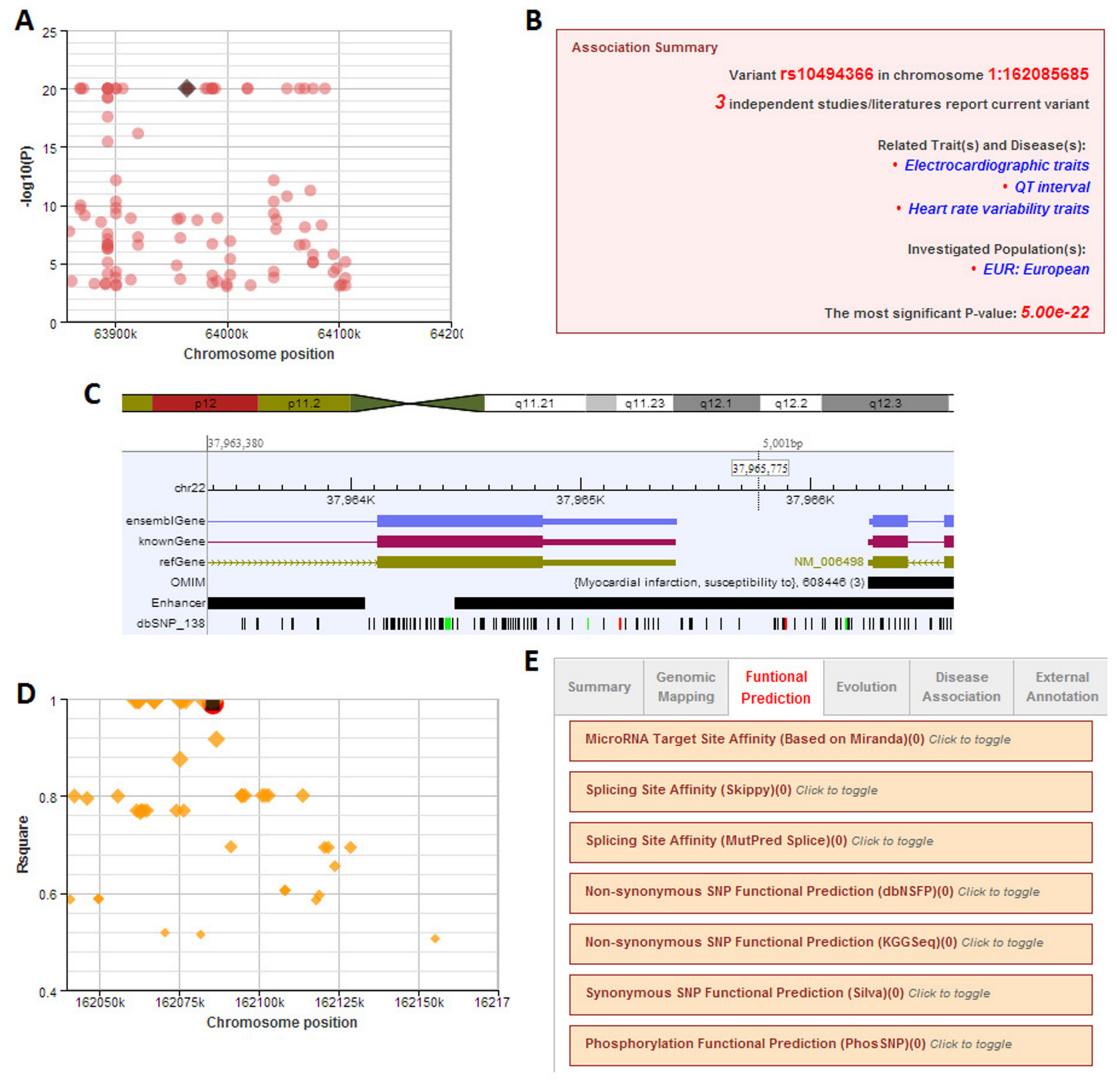
Main functions of GWASdb v2. (**A**) Interactive Manhattan panel; (**B**) TAS summary information; (**C**) Genome browser to show important functional elements; (**D**) Interactive LD panel; (**E**) GWASdb v2 annotation tabs.

GWASdb v2 provides four types of query entries (namely dbSNP ID, gene symbol, chromosome region, and trait name) for users to quickly inspect TASs of interest. It also offers a batch query function to allow users to upload a SNP list containing either SNP IDs or genomic coordinates. A query job will run in the backend, and users can provide their emails or keep the job URL to retrieve their results, which is downloadable and contains detailed GWAS information and SNP annotation. In the front page of GWASdb v2, a circos Manhattan plot shows summary of the latest GWASdb v2 TASs in the whole genome. Users can also enter into the single chromosome view by clicking corresponding chromosome cytoband. The basic statistics of GWASdb v2 are shown in the right tab of the front page, such as regional distribution and SNP type distribution in genic region. We improved the GWASdb v2 result page and made it more compact and integrative compared with previous version. We used an interactive panel to display the TASs in region of interest, users can perform moving, zooming and clicking operations to visualize the region within this panel (Figure 1A). The right tab panel of the result page summarizes the association information of current TAS including variant locus, the number of independent GWASs, related traits/diseases and populations (Figure 1B). There is also a table list to show detailed association information for each study. We incorporated a genome browser, where users can easily check surrounding genomic features for queried TAS such as RefGene, EnsembleGene, KnownGene, OMIM disease gene, and regulatory enhancer (Figure 1C). To display the annotation of queried TAS, GWASdb v2 uses a separate window to present comprehensive information by clicking the ‘Annotate Current Variant’ button. The LD panel shows the correlated SNPs for the queried leading TAS. Users can also change the LD reference and investigated population (eleven HapMap populations or four 1000 Genomes Project populations) (Figure 1D). Annotation information is classified into six major categories in interactive multi-tabs, including TAS summary, genomic elements, functional prediction, evolution, disease related evidence, as well as several convenient external links to DMDM (47), SNPedia, Regulomedb (48), HaploReg (49), rSNPBase (50), UCSC Genome Browser (51) and GWAS Central (Figure 1E).

**Figure 2.**
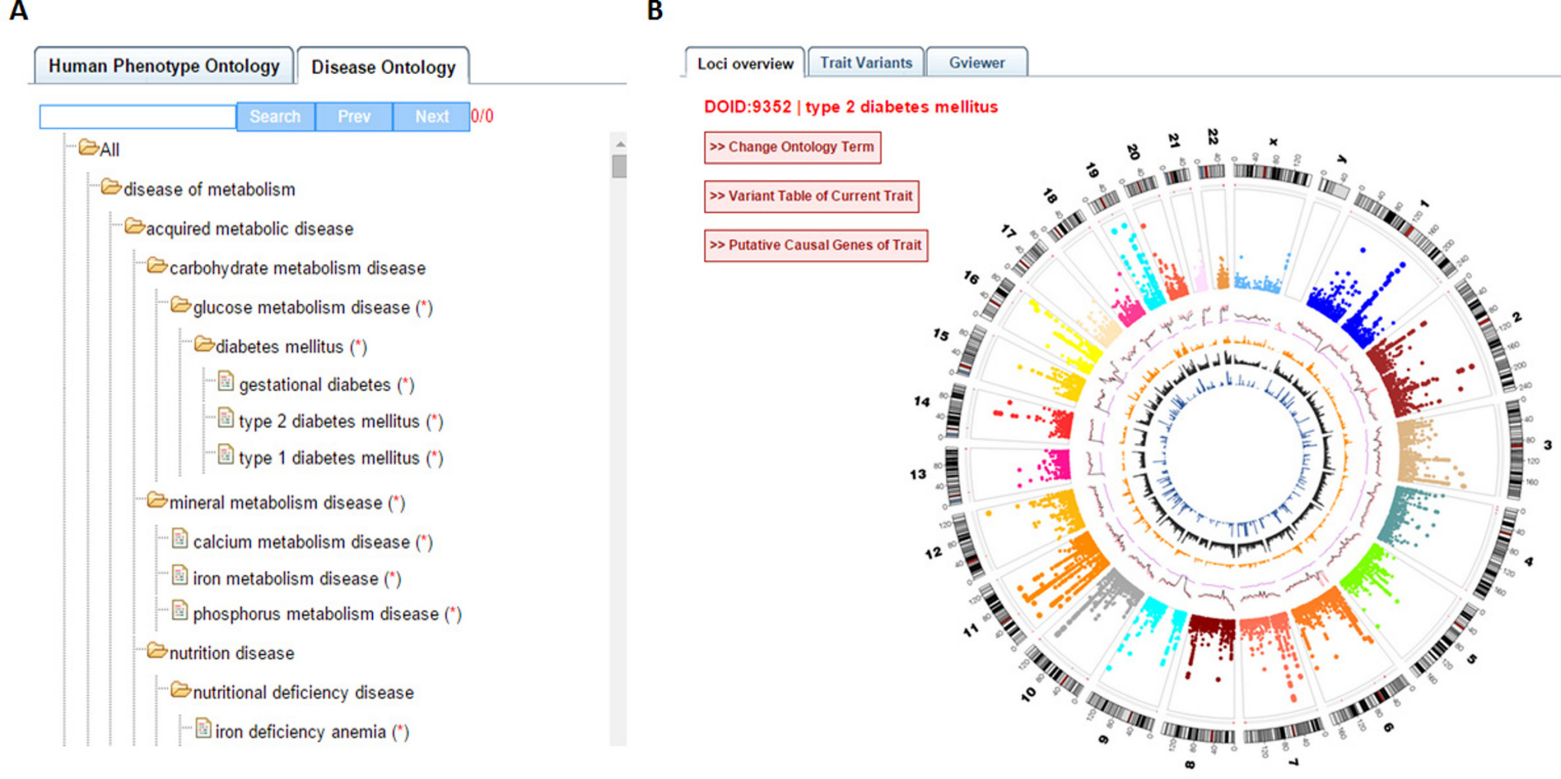
Trait/disease mapping interface in GWASdb v2. (**A**) Ontology tree viewer; (**B**) Genomic overview of SNPs associated with a particular disease.

To simplify the visualization of trait/disease ontology mapping, we embedded the whole ontology of HPO and DO to one page coupled with query function by the tree viewer and search box (Figure 2A). To query a particular trait, users could click the ‘Check Selected Trait’ button. In the particular trait page, there are three tabs: ‘Loci overview’, ‘Trait variants’ and ‘Gviewer’. In ‘Loci overview’, an interactive circos plot of all the SNPs associated with that trait across the genome is shown (Figure 2B). Users could further (i) check TASs on chromosome of interest by clicking on corresponding circle band; (ii) see TASs in a table view by clicking ‘Variant Table of Current Trait’ button; (iii) obtain putative causal genes in a table by clicking the ‘Putative Causal Genes of Trait’ button; or (iv) switch to other ontology terms by simply clicking on ‘Change Ontology Term’ button. Tab ‘Trait variants’ is available for individual TAS check in a chromosome-based Manhattan panel (Figure 1A). Tab ‘Gviewer’ provides function elements in a chromosome-based genome track view (Figure 1C). We also provided a ‘GWAS Dictionary’ function which allows users to browse trait/disease in a dictionary manner. A table within the browser will present query results such as genomic position, PubMed ID and *P*-values.

GWASdb v2 TASs, ontology mapping and annotation table can be downloaded via FTP (ftp://jjwanglab.org/GWASdb/) or remotely retrieved by Tabix. Besides, we compiled a number of RESTful interfaces to quickly redirect users to their interested SNPs, genes, regions or traits.

## DISCUSSION

In the past few years, GWASs have discovered a large number of new genetic loci associated with different traits/diseases in different human populations by incorporating larger cohorts, meta-analysis and cross-phenotype investigation. Since our last publication version of GWASdb, additional 1540 GWASs have been published and uncovered 105 993 new associations. As the cost of NGS is continuously reduced, large-scale whole exome sequencing (WES) and whole genome sequencing (WGS), which possess more advantages than GWAS chips to detect low frequency disease-associated variants, are currently applied to decipher the genetic association on many complex diseases (52) and will significantly overcome the issue of ‘missing heritability’ (53). During this stage, we have constantly updated GWASdb and released five major versions in the past three years.

Identification of the true causal and functional variants from a GWAS leading SNP is usually a tough work, which requires expensive and time-consuming experiments. Even though a few statistical methods can facilitate the fine mapping of true causal hits (4,54,55), it still lacks functional evidence to illustrate the role of these variants in biological mechanisms, and necessarily requires in-depth investigation. To this end, GWASdb v2 provides well-organized and comprehensive annotations for each TAS in various perspectives from gene-based information to knowledge-based functional prediction. Users can easily visualize their interested SNPs or traits interactively. Although UCSC Genome Browser and Variant Effect Predictor (VEP) (56) have provided variant annotations on multiple levels, GWASdb v2 further compiles functional predictions for TASs from different biological domains using prevalent tools. These predictions and annotations could serve as a valid resource to prioritize functional variants.

GWASdb now not only focuses on collection of TASs with significant or moderate effects, but also pays more attention on comprehensive annotation and prioritization of these variants. A batch of algorithms can accurately predict the deleteriousness of non-synonymous mutations that directly alter protein sequences, but for other types of mutations such as variants in regulatory regions, effective algorithms and statistical methods are still in urgent need. Recent works combine multiple genomic data in scoring and prioritizing functional non-coding variants (57–60), however, positive datasets and systematic solutions to validate the prediction power are insufficient (61). In addition to constant collection of new TASs, one of the major tasks for GWASdb in the future is to fine-map and exploit the true functional variants which are causal for disease development and abnormal phenotypes.

## References

[1] McCarthy M.I., Abecasis G.R., Cardon L.R., Goldstein D.B., Little J., Ioannidis J.P., Hirschhorn J.N. (2008). Genome-wide association studies for complex traits: consensus, uncertainty and challenges. Nat. Rev. Genet..

[2] Stranger B.E., Stahl E.A., Raj T. (2011). Progress and promise of genome-wide association studies for human complex trait genetics. Genetics.

[3] Rietveld C.A., Medland S.E., Derringer J., Yang J., Esko T., Martin N.W., Westra H.J., Shakhbazov K., Abdellaoui A., Agrawal A. (2013). GWAS of 126,559 individuals identifies genetic variants associated with educational attainment. Science.

[4] Spain S.L., Barrett J.C. (2015). Strategies for fine-mapping complex traits. Hum. Mol. Genet..

[5] Lambert J.C., Ibrahim-Verbaas C.A., Harold D., Naj A.C., Sims R., Bellenguez C., DeStafano A.L., Bis J.C., Beecham G.W., Grenier-Boley B. (2013). Meta-analysis of 74,046 individuals identifies 11 new susceptibility loci for Alzheimer's disease. Nat. Genet..

[6] Solovieff N., Cotsapas C., Lee P.H., Purcell S.M., Smoller J.W. (2013). Pleiotropy in complex traits: challenges and strategies. Nat. Rev. Genet..

[7] Saxena R., Elbers C.C., Guo Y., Peter I., Gaunt T.R., Mega J.L., Lanktree M.B., Tare A., Castillo B.A., Li Y.R. (2012). Large-scale gene-centric meta-analysis across 39 studies identifies type 2 diabetes loci. Am. J. Hum. Genet..

[8] Blickwedehl J., Olejniczak S., Cummings R., Sarvaiya N., Mantilla A., Chanan-Khan A., Pandita T.K., Schmidt M., Thompson C.B., Bangia N. (2012). The proteasome activator PA200 regulates tumor cell responsiveness to glutamine and resistance to ionizing radiation. Mol. Cancer Res.: MCR.

[9] Amberger J.S., Bocchini C.A., Schiettecatte F., Scott A.F., Hamosh A. (2015). OMIM.org: Online Mendelian Inheritance in Man (OMIM(R)), an online catalog of human genes and genetic disorders. Nucleic Acids Res..

[10] Nelson M.R., Tipney H., Painter J.L., Shen J., Nicoletti P., Shen Y., Floratos A., Sham P.C., Li M.J., Wang J. (2015). The support of human genetic evidence for approved drug indications. Nat. Genet..

[11] Welter D., MacArthur J., Morales J., Burdett T., Hall P., Junkins H., Klemm A., Flicek P., Manolio T., Hindorff L. (2014). The NHGRI GWAS Catalog, a curated resource of SNP-trait associations. Nucleic Acids Res..

[12] Ramos E.M., Hoffman D., Junkins H.A., Maglott D., Phan L., Sherry S.T., Feolo M., Hindorff L.A. (2014). Phenotype-Genotype Integrator (PheGenI): synthesizing genome-wide association study (GWAS) data with existing genomic resources. Eur. J. Hum. Genet.: EJHG.

[13] Beck T., Hastings R.K., Gollapudi S., Free R.C., Brookes A.J. (2014). GWAS Central: a comprehensive resource for the comparison and interrogation of genome-wide association studies. Eur. J. Hum. Genet.: EJHG.

[14] Cariaso M., Lennon G. (2012). SNPedia: a wiki supporting personal genome annotation, interpretation and analysis. Nucleic Acids Res..

[15] Eicher J.D., Landowski C., Stackhouse B., Sloan A., Chen W., Jensen N., Lien J.P., Leslie R., Johnson A.D. (2015). GRASP v2.0: an update on the Genome-Wide Repository of Associations between SNPs and phenotypes. Nucleic Acids Res..

[16] Consortium E.P. (2012). An integrated encyclopedia of DNA elements in the human genome. Nature.

[17] Bernstein B.E., Stamatoyannopoulos J.A., Costello J.F., Ren B., Milosavljevic A., Meissner A., Kellis M., Marra M.A., Beaudet A.L., Ecker J.R. (2010). The NIH roadmap epigenomics mapping consortium. Nat. Biotechnol..

[18] Karchin R. (2009). Next generation tools for the annotation of human SNPs. Brief. Bioinformatics.

[19] Li M.J., Sham P.C., Wang J. (2012). Genetic variant representation, annotation and prioritization in the post-GWAS era. Cell Res..

[20] Pabinger S., Dander A., Fischer M., Snajder R., Sperk M., Efremova M., Krabichler B., Speicher M.R., Zschocke J., Trajanoski Z. (2014). A survey of tools for variant analysis of next-generation genome sequencing data. Brief. Bioinformatics.

[21] Malone J., Holloway E., Adamusiak T., Kapushesky M., Zheng J., Kolesnikov N., Zhukova A., Brazma A., Parkinson H. (2010). Modeling sample variables with an Experimental Factor Ontology. Bioinformatics.

[22] Lipscomb C.E. (2000). Medical Subject Headings (MeSH). Bull. Med. Library Assoc..

[23] Groza T., Kohler S., Moldenhauer D., Vasilevsky N., Baynam G., Zemojtel T., Schriml L.M., Kibbe W.A., Schofield P.N., Beck T. (2015). The human phenotype ontology: semantic unification of common and rare disease. Am. J. Hum. Genet..

[24] Yu W., Gwinn M., Clyne M., Yesupriya A., Khoury M.J. (2008). A navigator for human genome epidemiology. Nat. Genet..

[25] Kibbe W.A., Arze C., Felix V., Mitraka E., Bolton E., Fu G., Mungall C.J., Binder J.X., Malone J., Vasant D. (2015). Disease Ontology 2015 update: an expanded and updated database of human diseases for linking biomedical knowledge through disease data. Nucleic Acids Res..

[26] Du P., Feng G., Flatow J., Song J., Holko M., Kibbe W.A., Lin S.M. (2009). From disease ontology to disease-ontology lite: statistical methods to adapt a general-purpose ontology for the test of gene-ontology associations. Bioinformatics.

[27] Tasan M., Musso G., Hao T., Vidal M., MacRae C.A., Roth F.P. (2015). Selecting causal genes from genome-wide association studies via functionally coherent subnetworks. Nat. Methods.

[28] Eichelbaum M., Ingelman-Sundberg M., Evans W.E. (2006). Pharmacogenomics and individualized drug therapy. Annu. Rev. Med..

[29] Law V., Knox C., Djoumbou Y., Jewison T., Guo A.C., Liu Y., Maciejewski A., Arndt D., Wilson M., Neveu V. (2014). DrugBank 4.0: shedding new light on drug metabolism. Nucleic Acids Res..

[30] Li M.J., Wang L.Y., Xia Z., Sham P.C., Wang J. (2013). GWAS3D: Detecting human regulatory variants by integrative analysis of genome-wide associations, chromosome interactions and histone modifications. Nucleic Acids Res..

[31] Bhattacharya A., Ziebarth J.D., Cui Y. (2014). PolymiRTS Database 3.0: linking polymorphisms in microRNAs and their target sites with human diseases and biological pathways. Nucleic Acids Res..

[32] Woolfe A., Mullikin J.C., Elnitski L. (2010). Genomic features defining exonic variants that modulate splicing. Genome Biol.

[33] Mort M., Sterne-Weiler T., Li B., Ball E.V., Cooper D.N., Radivojac P., Sanford J.R., Mooney S.D. (2014). MutPred Splice: machine learning-based prediction of exonic variants that disrupt splicing. Genome Biol..

[34] Liu X., Jian X., Boerwinkle E. (2013). dbNSFP v2.0: a database of human non-synonymous SNVs and their functional predictions and annotations. Hum. Mutat..

[35] Li M.J., Wang L.Y., Xia Z., Wong M.P., Sham P.C., Wang J. (2014). dbPSHP: a database of recent positive selection across human populations. Nucleic Acids Res..

[36] Pollard K.S., Hubisz M.J., Rosenbloom K.R., Siepel A. (2010). Detection of nonneutral substitution rates on mammalian phylogenies. Genome Res..

[37] Davydov E.V., Goode D.L., Sirota M., Cooper G.M., Sidow A., Batzoglou S. (2010). Identifying a high fraction of the human genome to be under selective constraint using GERP++. PLoS Comput. Biol..

[38] Becker K.G., Barnes K.C., Bright T.J., Wang S.A. (2004). The genetic association database. Nat. Genet..

[39] Landrum M.J., Lee J.M., Riley G.R., Jang W., Rubinstein W.S., Church D.M., Maglott D.R. (2014). ClinVar: public archive of relationships among sequence variation and human phenotype. Nucleic Acids Res..

[40] Forbes S.A., Beare D., Gunasekaran P., Leung K., Bindal N., Boutselakis H., Ding M., Bamford S., Cole C., Ward S. (2015). COSMIC: exploring the world's knowledge of somatic mutations in human cancer. Nucleic Acids Res..

[41] Li H. (2011). Tabix: fast retrieval of sequence features from generic TAB-delimited files. Bioinformatics.

[42] Krzywinski M., Schein J., Birol I., Connors J., Gascoyne R., Horsman D., Jones S.J., Marra M.A. (2009). Circos: an information aesthetic for comparative genomics. Genome Res..

[43] Juan L., Teng M., Zang T., Hao Y., Wang Z., Yan C., Liu Y., Li J., Zhang T., Wang Y. (2014). The personal genome browser: visualizing functions of genetic variants. Nucleic Acids Res..

[44] Schaub M.A., Boyle A.P., Kundaje A., Batzoglou S., Snyder M. (2012). Linking disease associations with regulatory information in the human genome. Genome Res..

[45] Nicolae D.L., Gamazon E., Zhang W., Duan S., Dolan M.E., Cox N.J. (2010). Trait-associated SNPs are more likely to be eQTLs: annotation to enhance discovery from GWAS. PLoS Genet..

[46] Grossman S.R., Andersen K.G., Shlyakhter I., Tabrizi S., Winnicki S., Yen A., Park D.J., Griesemer D., Karlsson E.K., Wong S.H. (2013). Identifying recent adaptations in large-scale genomic data. Cell.

[47] Peterson T.A., Adadey A., Santana-Cruz I., Sun Y., Winder A., Kann M.G. (2010). DMDM: domain mapping of disease mutations. Bioinformatics.

[48] Boyle A.P., Hong E.L., Hariharan M., Cheng Y., Schaub M.A., Kasowski M., Karczewski K.J., Park J., Hitz B.C., Weng S. (2012). Annotation of functional variation in personal genomes using RegulomeDB. Genome Res..

[49] Ward L.D., Kellis M. (2012). HaploReg: a resource for exploring chromatin states, conservation, and regulatory motif alterations within sets of genetically linked variants. Nucleic Acids Res..

[50] Guo L., Du Y., Chang S., Zhang K., Wang J. (2014). rSNPBase: a database for curated regulatory SNPs. Nucleic Acids Res..

[51] Karolchik D., Barber G.P., Casper J., Clawson H., Cline M.S., Diekhans M., Dreszer T.R., Fujita P.A., Guruvadoo L., Haeussler M. (2014). The UCSC Genome Browser database: 2014 update. Nucleic Acids Res..

[52] Rabbani B., Tekin M., Mahdieh N. (2014). The promise of whole-exome sequencing in medical genetics. J. Hum. Genet..

[53] Manolio T.A., Collins F.S., Cox N.J., Goldstein D.B., Hindorff L.A., Hunter D.J., McCarthy M.I., Ramos E.M., Cardon L.R., Chakravarti A. (2009). Finding the missing heritability of complex diseases. Nature.

[54] Faye L.L., Machiela M.J., Kraft P., Bull S.B., Sun L. (2013). Re-ranking sequencing variants in the post-GWAS era for accurate causal variant identification. PLoS Genet..

[55] Yang J., Ferreira T., Morris A.P., Medland S.E., Genetic Investigation of A.T.C., Replication D.I.G., Meta-analysis C., Madden P.A., Heath A.C., Martin N.G. (2012). Conditional and joint multiple-SNP analysis of GWAS summary statistics identifies additional variants influencing complex traits. Nat. Genet..

[56] McLaren W., Pritchard B., Rios D., Chen Y., Flicek P., Cunningham F. (2010). Deriving the consequences of genomic variants with the Ensembl API and SNP Effect Predictor. Bioinformatics.

[57] Kircher M., Witten D.M., Jain P., O'Roak B.J., Cooper G.M., Shendure J. (2014). A general framework for estimating the relative pathogenicity of human genetic variants. Nat. Genet..

[58] Li M.J., Wang J. (2015). Current trend of annotating single nucleotide variation in humans–A case study on SNVrap. Methods.

[59] Pickrell J.K. (2014). Joint analysis of functional genomic data and genome-wide association studies of 18 human traits. Am. J. Hum. Genet..

[60] Ritchie G.R., Dunham I., Zeggini E., Flicek P. (2014). Functional annotation of noncoding sequence variants. Nat. Methods.

[61] Li M.J., Yan B., Sham P.C., Wang J. (2014). Exploring the function of genetic variants in the non-coding genomic regions: approaches for identifying human regulatory variants affecting gene expression. Brief. Bioinformatics.

